# Epidemiology and evolution of Middle East respiratory syndrome coronavirus, 2012–2020

**DOI:** 10.1186/s40249-021-00853-0

**Published:** 2021-05-08

**Authors:** An-Ran Zhang, Wen-Qiang Shi, Kun Liu, Xin-Lou Li, Ming-Jin Liu, Wen-Hui Zhang, Guo-Ping Zhao, Jin-Jin Chen, Xiao-Ai Zhang, Dong Miao, Wei Ma, Wei Liu, Yang Yang, Li-Qun Fang

**Affiliations:** 1 Department of Epidemiology, School of Public Health, Cheeloo College of Medicine, Shandong University, 44 West Wenhua Road, Jinan, People’s Republic of China; 2 State Key Laboratory of Pathogen and Biosecurity, Beijing Institute of Microbiology and Epidemiology, 20 Dong-Da Street, Fengtai District, Beijing, 100071 People’s Republic of China; 3 Department of Biostatistics, College of Public Health and Health Professions, and Emerging Pathogens Institute, University of Florida, Gainesville, FL USA; 4 Department of Epidemiology, Ministry of Education Key Lab of Hazard Assessment and Control in Special Operational Environment, School of Public Health, Air Force Medical University, Xi’an, People’s Republic of China; 5 Department of Medical Research, Key Laboratory of Environmental Sense Organ Stress and Health of the Ministry of Environmental Protection, PLA Stragetic Support Force Characteristic Medical Center, Beijing, People’s Republic of China; 6 Logistics College of Chinese People’s Armed Police Forces, Tianjin, People’s Republic of China

**Keywords:** Middle East respiratory syndrome, MERS-CoV, Case fatality rate, Spatial diffusion, Phylogeny, Phylogeographic dynamic

## Abstract

**Background:**

The ongoing transmission of the Middle East respiratory syndrome coronavirus (MERS-CoV) in the Middle East and its expansion to other regions are raising concerns of a potential pandemic. An in-depth analysis about both population and molecular epidemiology of this pathogen is needed.

**Methods:**

MERS cases reported globally as of June 2020 were collected mainly from World Health Organization official reports, supplemented by other reliable sources. Determinants for case fatality and spatial diffusion of MERS were assessed with Logistic regressions and Cox proportional hazard models, respectively. Phylogenetic and phylogeographic analyses were performed to examine the evolution and migration history of MERS-CoV.

**Results:**

A total of 2562 confirmed MERS cases with 150 case clusters were reported with a case fatality rate of 32.7% (95% *CI*: 30.9‒34.6%). Saudi Arabia accounted for 83.6% of the cases. Age of ≥ 65 years old, underlying conditions and ≥ 5 days delay in diagnosis were independent risk factors for death. However, a history of animal contact was associated with a higher risk (adjusted *OR* = 2.97, 95% *CI*: 1.10–7.98) among female cases < 65 years but with a lower risk (adjusted *OR* = 0.31, 95% *CI*: 0.18–0.51) among male cases ≥ 65 years old. Diffusion of the disease was fastest from its origin in Saudi Arabia to the east, and was primarily driven by the transportation network. The most recent sub-clade C5.1 (since 2013) was associated with non-synonymous mutations and a higher mortality rate. Phylogeographic analyses pointed to Riyadh of Saudi Arabia and Abu Dhabi of the United Arab Emirates as the hubs for both local and international spread of MERS-CoV.

**Conclusions:**

MERS-CoV remains primarily locally transmitted in the Middle East, with opportunistic exportation to other continents and a potential of causing transmission clusters of human cases. Animal contact is associated with a higher risk of death, but the association differs by age and sex. Transportation network is the leading driver for the spatial diffusion of the disease. These findings how this pathogen spread are helpful for targeting public health surveillance and interventions to control endemics and to prevent a potential pandemic.

**Graphical abstract:**

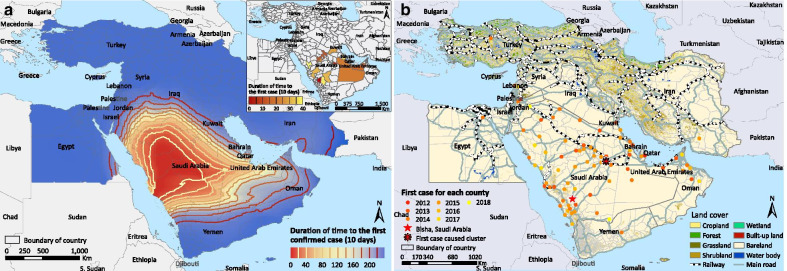

**Supplementary Information:**

The online version contains supplementary material available at 10.1186/s40249-021-00853-0.

## Background

Middle East respiratory syndrome (MERS) is a respiratory infectious disease first discovered in the Kingdom of Saudi Arabia in September 2012 [1]. The disease is caused by the Middle East respiratory syndrome coronavirus (MERS-CoV) which can be highly pathogenic in humans. Individuals infected with MERS-CoV may experience none, mild or severe respiratory illnesses or even death. As of 30 May 2020, a total of 27 countries in the Middle East, North Africa, Europe, Northeast Asia, and North America have reported 2562 laboratory-confirmed MERS cases and 881 associated deaths, according to the World Health Organization (WHO) [2]. The vast majority of MERS cases were reported by the Saudi Arabia, followed by Republic of Korea [2]. Frequent travelers and worshippers from and to the Middle East have raised the concern about a global pandemic, given the lack of effective treatment and vaccine [3]. In February 2018, WHO formally incorporated MERS into the Research and Development Blueprint (the R&D Blueprint) to promote research in this area [4].

Current epidemiological studies suggest that human-to-human transmission of MERS-CoV is inefficient, and the primary infection mode is via direct/indirect contact with dromedary camels, although other mammals may also serve as the reservoir [5–10]. On the other hand, human clusters of MERS have been continuously observed in healthcare and household settings, especially among people with chronic conditions or compromised immunity [11, 12]. Several studies explored risk factors for the transmission of MERS-CoV at the individual level in specific countries and found that infection risk was mainly driven by recent exposure to dromedary or its raw products, chronic conditions, or close contact with other MERS patients [11, 13–16]. However, very few studies have systematically analyzed spatial diffusion of the virus at the population level and associated risk factors. In addition, while it has been shown that chronic condition and male sex are highly predictive of fatal outcomes [17], no study has examined potential interactions among key predictors for death, e.g., demographic characteristics and animal contact. Some of these predictors are correlated, e.g., males tend to have much more frequent contact with dromedary. Consequently, it is necessary to condition on one predictor when evaluating the effect of another.

In the midst of the pandemic of severe acute respiratory syndrome coronavirus 2 (SARS-CoV-2), it is crucial to understand the epidemiological characteristics and evolutionary history for MERS-CoV as the two coronaviruses are genetically related. The possibility of recombination between the two viruses if co-infection of the same host occurs cannot be totally ruled out, as their host species do overlap, e.g., humans and bats [18]. Thus far, phylogenetic and phylogeographic analyses focusing on MERS-CoV have been either outdated or restricted to small data sets [9, 10, 19–21]. In addition, some evolutionary characteristics of MERS-CoV such as which genes are subject to positive selection, need to be closely monitored.

By assembling MERS surveillance and contact tracing data up to June of 2020 from public health agencies and peer-reviewed literature, we summarized the epidemiological features and spatiotemporal spread of MERS around the globe. We investigated risk factors for fatality and how their effects could be modified by each other. In addition, we assessed the roles of a variety of environmental, socioeconomic, and biological factors in the spatial diffusion of MERS-CoV. Using publicly available MERS-CoV full-genome sequences, we further assessed the evolution and migration history of the virus. Piecing these results together, we aim to provide an up-to-date picture about both population and molecular epidemiology of this pathogen.

## Method

### Data collection and management

We assembled three datasets: (1) a list of individual human cases worldwide with demographic, exposure and clinical information, (2) eco-geographic and socioeconomic characteristics (referred to as socioenvironmental variables hereinafter) at the appropriate administrative level in the Middle East (county for Saudi Arabia and province for the other countries), and (3) full-genome sequences of MERS-CoV worldwide.

Data on confirmed MERS cases were collected from official reports of WHO, the Food and Agriculture Organization (FAO) of the United Nations, and the health departments of affected countries, which were cross-validated with and supplemented by data from websites and literatures. Search medical subject headings (MeSH) terms used were “Middle East Respiratory Syndrome Coronavirus/MERS-CoV/HCoV-EMC” or “Middle East respiratory syndrome/Middle Eastern Respiratory Syndrome/MERS”.

All the cases had been confirmed following a standard WHO technical guidance (https://www.who.int/csr/disease/coronavirus_infections/case_definition/en/). A valid record of MERS case must include the basic demographic information (gender, age, reporting country, city of residence, being healthcare worker or not, baseline chronic conditions), dates of critical events (such as symptom onset, first hospitalization, laboratory confirmation), and exposure information (whether exposed to animal or its raw production, or exposed to confirmed MERS patients). Cases without any individual information, duplicated records and no-confirmed cases were removed. Each confirmed case was geo-referenced and mapped according to the finest address available using GIS technologies. Thematic maps of cumulative numbers of confirmed MERS cases and clusters were created using ArcGIS 10.5 (Esri Inc, Redlands, CA, USA).

The following socioenvironmental variables potentially related to the transmission of MERS were collected: population density, camel density, monthly meteorological data, elevation, land cover, economic development level, transportation, locations of hospitals. Full-genome sequences (> 30 000 bp) of MERS-CoV up to June of 2020, together with isolation year, host type and location, were retrieved from GenBank. Protein and the coding sequences (CDS) sequences of these MERS-CoV sequences were derived. All the data sources (Additional file 1: Table S1) involved in this study were introduced in detail in the Additional file 1: Additional Appendix.

### Descriptive analysis

MERS-affected countries were grouped into four categories of transmission type: (i) zoonotic transmission plus human-to-human transmission, (ii) zoonotic transmission without human-to-human transmission, (iii) imported infection plus human-to-human transmission, and (iv) imported infection without human-to-human transmission (Additional file 1: Table S2). Existence of zoonotic transmission was determined by reported endemic circulation with frequent zoonotic introduction. A case cluster is defined as a group of two or more epidemiologically linked cases, where epidemiological link refers to close contact as indicated by the source database or literature. For example, the WHO database provides the ID number of the source case for some cases. By this definition, all cases in the outbreak in Republic of Korea can be traced to the same imported case and are therefore considered a single case cluster [13]. Demographic characteristics were compared between country categories and between case types using the Kruskal–Wallis test for continuous variables and the *χ*^*2*^ test for categorical variables.

### Analysis of risk factors for cases fatality

Logistic regression was performed to explore risk factors associated with the survival outcome of MERS cases. All MERS cases with reliable survival outcome and contact information (before symptom onset) were included in the analysis. The following potential risk factors were included in the analysis: age group (≥ 65 years old vs < 65 years old), sex (male vs female), region (Middle East vs other), occupation (healthcare worker vs other), underlying chronic conditions including diabetes mellitus, renal failure, chronic respiratory and circulatory diseases and compromised immune systems (yes, no, unknown), animal contact (yes, no, unknown), time from disease onset to confirmation (OTC, > 5 days vs ≤ 5 days), and reporting year (2012–2014, 2015–2016, 2017–2020). Among variables with missing values, OTC were imputed using R package “*mice*”. But missing values of underlying condition and animal contact were treated into a category of unknown, for imputation efficiency is not high for variables with a missing rate over 30%, Backward elimination was used to a parsimonious model (threshold *P*-value = 0.05). Two-way interactions among age group, sex and animal contact were considered based on the amount of data per category of each factor.

### Spatiotemporal diffusion

We limited the spatiotemporal diffusion analysis to the Middle East Region, the main endemic region of MERS. Opportunistic long-distance exportations of MERS cases, e.g., to Europe and Republic of Korea, were not considered. The spatial unit used in this study is the second-level administrative unit, e.g., province, for most countries. Provinces of Saudi Arabia are much larger than those of neighboring countries. To make spatial units comparable between countries, we use the third-level administrative area (county) as the spatial unit for Saudi Arabia. Finally, a total of 283 administrative units were included in the spatiotemporal diffusion analysis.

We obtained 34 socioenvironmental variables from various data sources (Additional file 1: Table S3). Camel density was available only at the national level in many countries. Considering the role of camel as a MERS-CoV reservoir, we imputed missing values of camel density using the R package “*mice*”. To account for uncertainties in these missing values, we generated 100 imputed sets of camel density and averaged all analyses related to camel density over these imputation sets. To reduce collinearity among these variables, we screened pairwise correlations. If two variables have an absolute Pearson correlation higher than 0.65, the one with a higher average correlation with all other variables was excluded.

A Cox proportional hazard model was used to assess which socioenvironmental variables were associated with the reporting time (since September 2012) of the first MERS case in each space unit. A spatial trend contour plot was developed to visualize the spatial diffusion of the disease. The time between adjacent contours was fixed at 200 days, and a wider gap between adjacent contours indicates a faster spatial diffusion. All descriptive and diffusion analyses were performed using the R software version 3.6.2 (R Foundation for Statistical Computing, Vienna, Austria). A two-sided *P*-value < 0.05 was considered statistically significant.

### Phylogenetic and phylogeographic analyses

The whole-genome sequences were analyzed using toolkits provided by the Nextstrain framework [22]. Sequences were aligned using MAFFT v7.407 [23], and the alignment was trimmed to a reference genome (GenBank accession ID: NC_019843.3). A phylogenetic tree was built using a maximum likelihood approach implemented in IQ-Tree v1.6.10 [24]. For the phylogeographic analysis, TreeTime was used to infer the divergence time, discrete traits of the ancestral nodes (location and host), and geographic transmission history across the tree [25]. To detect sites under positive selection among CDS of protein genes, for each gene, the original open reading frame (ORF) sequences were first aligned using MAFFT and CDS were aligned using PAL2NAL v14 under the guidance for protein alignment [26]. CodeML in PAML v4.9 as part of the ETE 3 package (v 3.1.1) was used to detect positive selection sites by branch-site test [27, 28]. To balance the sample size of each host species, human and camel sequences were randomly down-sampled to five sequences per host species. After smoothing mortality rate and incidence rate over space and time, we matched these smoothed rates with tree tips (MERS-CoV sequences) by specimen collection year and location to assess potential association of phylogeny with mortality and incidence rates.

## Results

### Epidemiological features of MERS cases

**Fig. 1:**
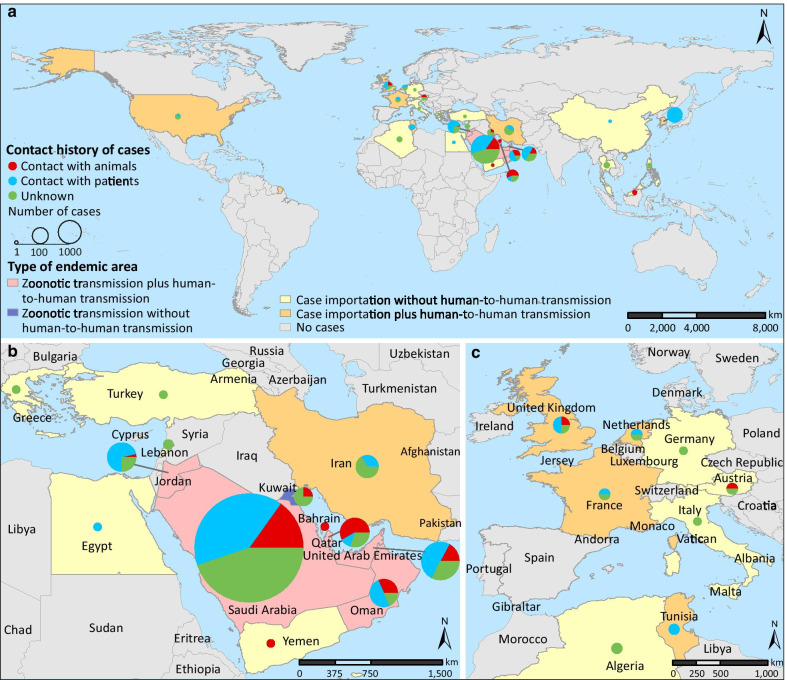
Distribution of human MERS cases in the world **a**, in the Middle East **b** and in Europe **c** during 2012–2020. Countries were colored according to the dominant transmission type: (**i**) zoonotic transmission plus human-to-human transmission, (**ii**) zoonotic transmission without human-to-human transmission, (**iii**) imported infection plus human-to-human transmission, and (**iv**) imported infection without human-to-human transmission.

From September 2012 to June 2020, a total of 2562 laboratory-confirmed MERS cases were reported. After excluding 112 cases with incomplete data, 2450 confirmed MERS cases, together with 150 derived case clusters, were included in subsequent analyses (Table 1). The median age was 53 years old (IQR: 38‒65), and 69.4% of cases were male. Healthcare workers accounted for 13.7% (335/2450) of the total patients. The median time from disease onset to diagnosis was 5 days (IQR: 3‒8). Death occurred in 802 patients, leading to a case fatality rate (CRF) of 32.7% (95% *CI*: 30.9‒34.6%). Among the 1453 patients with known exposure history, 356 (24.0%) reported animal contact. Zoonotic infections (i.e., countries in transmission categories i and ii) only occurred in the Middle East, although cases with animal contact had also been imported into Europe and Southeast Asia (Fig. 1). Among countries with locally infected patients, the proportion of cases with animal contact exceeded 50% in Qatar, followed by 15‒29% in Oman, United Arab Emirates (UAE), and Saudi Arabia (Fig. 1).

**Table 1:** Baseline demographic and clinical characteristics of confirmed Middle East respiratory syndrome cases reported between September 2012 and June 2020

	Number (%) of cases				
	Total	Saudi Arabia	Korea	United Arab Emirates	Others
Number of confirmed cases	2450	2048	186	93	123
Annual incidence (/10^7^)	0.45	94.34	4.71	14.07	0.023
Female	751 (30.7)	626 (30.6)	75 (40.3)	20 (21.5)	30 (24.4)
Age, years (median, IQR)	53 (38–65)	53 (38–65)	55 (42–66)	43 (33–59)	52 (38–65)
Number of case cluster	150	123	1	11	15
Number of deaths (CFR, %)	802 (32.7)	718 (35.1)	35 (18.8)	15 (16.1)	34 (27.6)
Contact history					
Animals	356 (14.5)	310 (15.1)	0	16 (17.2)	30 (24.4)
Patients	1097 (44.8)	820 (40.1)	184 (98.9)	47 (50.5)	46 (37.4)
Unknown	997 (40.7)	918 (44.8)	2 (1.1)	30 (32.3)	47 (38.2)
Occupation					
Healthcare worker	335 (13.7)	265 (12.9)	30 (16.1)	26 (28.0)	14 (11.4)
Others	2115 (86.3)	1783 (87.1)	156 (83.9)	67 (72.0)	109 (88.6)
Exposure site					
Hospital	724 (29.6)	496 (24.2)	185 (99.5)	21 (22.6)	22 (17.9)
Household	120 (4.9)	93 (4.5)	0	11 (11.8)	16 (13.0)
Others	1606 (65.5)	1459 (71.3)	1 (0.5)	61 (65.6)	85 (69.1)
Asymptomatic infection	201 (8.2)	173 (8.5)	0	19 (20.4)	9 (7.3)
Underlying condition	1272 (51.9)	1156 (56.5)	20 (10.8)	38 (40.9)	58 (47.2)
Time from disease onset to diagnosis, days (median, IQR)	5 (3–8)	5 (3–8)	4 (2–8)	8 (6–14)	8 (5–13.5)
Time from disease onset to death, days (median, IQR)	11 (7–17)	10 (6–16)	12 (9–16)	21.5 (18–27)	16 (11–20)
Year of occurrence					
2012	9 (0.4)	5 (0.2)	0	0	4 (3.3)
2013	169 (6.9)	137 (6.7)	0	12 (12.9)	20 (16.3)
2014	652 (26.6)	560 (27.3)	0	59 (63.3)	33 (26.8)
2015	680 (27.8)	457 (22.3)	185 (99.5)	7 (7.5)	31 (25.2)
2016	254 (10.4)	242 (11.8)	0	3 (3.2)	9 (7.3)
2017	250 (10.2)	237 (11.6)	0	7 (7.5)	6 (4.9)
2018	150 (6.1)	145 (7.1)	1 (0.5)	1 (1.2)	3 (2.4)
2019	226 (9.2)	208 (10.2)	0	2 (2.2)	16 (13.0)
2020	60 (2.4)	57 (2.8)	0	2 (2.2)	1 (0.8)

IQR, interquartile range; CFR, case fatality rate

Saudi Arabia, Republic of Korea and UAE together reported the vast majority of the cases and case clusters (Additional file 1: Table S2 and Figure S1‒S2). Patients from the three countries shared similar age and sex distributions, but Saudi Arabia had the highest CFR (35.1%), probably due to a higher proportion of underlying conditions (Table 1). Republic of Korea had the highest proportion (> 99%) of hospital infections and the shortest time (median = 4 days) from disease onset to diagnosis, yet its CFR (18.8%) is comparable to that of UAE (16.1%). UAE had the highest proportion of asymptomatic infections and the longest survival time among fatal cases (Table 1).

Cases with animal contact were older, more likely to be male, and more likely to have underlying conditions and a longer delay from disease onset to diagnosis, in comparison to cases without (Additional file 1: Table S4). These characteristics of cases with animal contact could partially explain their significantly higher CFR (35.1% vs 24.3%, *P* < 0.001) than those without animal contact (Additional file 1: Table S4). However, among fatal cases, those with animal contact had a slightly longer survival time than those without (median = 11.5 vs 9 days, *P* < 0.001). Cases with only patient contact were more likely to be health-care workers and more likely to be asymptomatic (Additional file 1: Table S4). The proportion of cases with animal contact was increasing from 2012 to 2018 and seemed to stabilize thereafter. Epidemics peaked mostly between April and September (Additional file 1: Figure S3). In contrast, cases with animal contact were more likely to occur from January to March. Seasonality differed slightly between countries, e.g., peaks of zoonotic infections in UAE were also observed in May. The peak of the global epidemic in June of 2015 was due to the opportunistic outbreak in Republic of Korea, which does not necessarily reflect seasonality in the endemic setting.

### Determinants for case fatality

All the eight individual-level factors (age group, sex, living in the Middle East, healthcare worker, underlying conditions, animal contact history before onset, delay from disease onset to diagnosis and onset year) were significantly associated with mortality in the univariate analysis, with age and underlying condition as the leading factors with *OR* > 5 (Table 2). We identified a multivariate model containing all eight main effects, as well as two-way interactions among age group, sex and animal contact. After controlling for other factors, underlying condition was associated with 3.5-fold increase in the risk (*OR* = 3.5, 95% *CI*: 2.5–4.89), whereas being a healthcare worker was protective (*OR* = 0.33, 95% *CI*: 0.21–0.54). OTC ≥ 5 days increased the risk by 31% (95% *CI*: 5–63%). Compared to 2012–2014, the risk of death was higher during 2015–2016 (*OR* = 1.63, 95% *CI*: 1.27–2.09) but lower during 2017–2019 (*OR* = 0.70, 95% *CI*: 0.53–0.92).

**Table 2:** Logistic regression analyses of potential risk factors for mortality among Middle East respiratory syndrome cases, September 2012–June 2020

		Outcome (*n*)		Univariable			Multivariable		
		Death/Total	CFR (95% *CI*)	*OR*	95% CI*	*P*-value*	Adjusted *OR*	95% CI*	*P*-value*
Age group	≥ 65 years	392/654	59.9 (58.0–61.9)	5.06	4.18–6.12	< 0.001	10.52	6.46–17.11	< 0.001
	< 65 years	410/1796	22.8 (21.2–24.5)	1.00	–	–	1.00	–	–
Sex	Male	604/1699	35.6 (33.7–37.5)	1.54	1.27–1.86	< 0.001	2.34	1.57–3.49	< 0.001
	Female	198/751	26.4 (24.6–28.1)	1.00	-	-–	1.00	–	–
Region	Middle East	761/2236	34.0 (32.1–36.0)	2.18	1.53–3.09	< 0.001	3.09	1.97–4.84	< 0.001
	Other	41/214	19.2 (13.9–24.4)	1.00	–	–	1.00	–	–
Healthcare worker	Yes	22/335	6.6 (3.9–9.2)	0.12	0.08–0.19	< 0.001	0.33	0.21–0.54	< 0.001
	No	780/2115	36.9 (34.8–38.9)	1.00	–	–	1.00	–	–
Underlying condition	Yes	587/1272	46.2 (43.4–48.9)	6.29	4.62–8.55	< 0.001	3.50	2.50–4.89	< 0.001
	No	53/442	12.0 (9.0–15.0)	1.00	–	–	1.00	–	–
	Unknown	162/736	22.0 (19.0–25.0)	2.07	1.48–2.90	< 0.001	1.92	1.31–2.81	0.001
Animal Contact	Yes	125/356	35.1 (30.2–40.1)	1.68	1.30–2.18	< 0.001	2.97	1.10–7.98	0.031
	No	267/1097	24.3 (21.8–26.9)	1.00	–	–	1.00	–	–
	Unknown	410/997	41.1 (38.1–44.2)	2.17	1.80–2.62	< 0.001	2.41	1.54–3.77	< 0.001
OTC^§^	> 5 days	331/679	48.8 (45.0–52.5)	1.34	1.11–1.62	0.003	1.31	1.05–1.63	0.019
	≤ 5 days	289/773	37.4 (34.0–40.8)	1.00	–	–	1.00	–	–
Year	2012–2014	256/830	30.8 (27.7–34.0)	1.00	–	–	1.00	–	–
	2015–2016	353/934	37.8 (34.7–40.9)	1.36	1.12–1.66	0.002	1.63	1.27–2.09	< 0.001
	2017–2019	193/686	28.1 (24.8–31.5)	0.88	0.70–1.10	0.25	0.70	0.53–0.92	0.010
Age group * Sex							0.54	0.33–0.87	0.012
Age group * Animal contact (with vs without)							0.29	0.16–0.53	< 0.001
Age group * Animal contact (unknown vs without)							0.45	0.28–0.72	0.001
Sex * Animal contact (with vs without)							0.36	0.13–0.97	0.043
Sex * Animal contact (unknown vs without)							0.58	0.36–0.94	0.028

*CFR* case fatality rate, *OR* odds ratio, *CI* confidence Interval, *OTC* Time from disease onset to confirmation

§OTC have missing values. Description (column 2–5) are based on non-missing values. But logistic analysis were based on data after interpolation

*Means that there must be no corresponding results for being the control groups

Odds ratios with regard to each of age group, sex and animal contact while controlling for the other two are shown in Additional file 1: Table S5–S7. The older age group (≥ 65 years) was associated with a higher risk of death in general, with significant *OR*s ranging 1.64–10.52 for all combinations of sex and animal contact history (Additional file 1: Table S5). However, the age effect was more prominent among patients without animal contact. Among patients without animal contact, the adjusted *OR* were 5.65 (95% *CI*: 3.75–8.50) for males and 10.52 (95% *CI*: 6.46–17.11) for females, compared to 1.64 (95% *CI*: 1.03–2.62) for males and 3.06 (95% *CI*: 1.59–5.86) for females among those with animal contact. From these estimates, it is also clear that the age effect was more prominent among female patients compared to male patients. Sex did not affect the risk of death significantly, although males tended to have higher risk among the younger age group without animal contact (adjusted *OR* = 2.34, 95% *CI*: 1.57–3.49, Additional file 1: Table S6). A history of animal contact was associated with a higher risk (adjusted *OR* = 2.97, 95% *CI*: 1.10–7.98) among female cases < 65 years but with a lower risk (adjusted *OR* = 0.31, 95% *CI*: 0.18–0.51) among male cases ≥ 65 years old (Additional file 1: Table S7). The model-estimated effects of age group, sex, and underlying conditions on CFR are in line with the observed CFRs stratified by each of these variables (Additional file 1: Figure S4). The CFR decreased gradually since 2015.

### Geographic expansion

After the screening of correlations, 20 out of the 34 socioenvironmental variables (Additional file 1: Table S3) remained for further analysis: population density, elevation, camel density, 8 variables regarding land covers (cropland, forest, shrubland, grassland, wetland, bareland, waterbody, urban), 6 ecoclimatic variables (bio1, bio2, bio3, bio4, bio5, bio12), transportation (railway, main road) and number of hospitals.

**Fig. 2:**
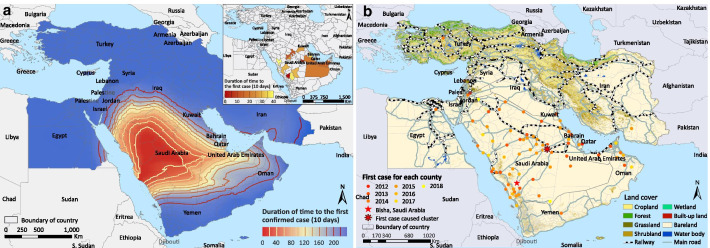
Geographic expansion of MERS **a** and its relationship with transportation network and land cover **b** in the Middle East. The first invasion time of each space unit was defined as the time lag between the first confirmed case for each unit and September 20, 2012, the symptom onset date of the first confirmed case in the Middle East Region. The time between adjacent contours was fixed at 200 days, and a wide gap between adjacent contours indicates a faster spatial diffusion of the disease. The inset map in panel **a** was added to present the early onset situation in the whole Middle East during the first 400 days.

The first case was reported in Bisha, central-west Saudi Arabia in September 2012. The disease spread more rapidly towards the east (UAE and Oman) than towards other directions (Fig. 2a). The diffusion appears to be acerating in recent years. At the second administrative level, 12 of the 14 eco-geographic variables were associated with the spread of MERS in the univariable Cox regressions (Table 3). In the multivariate analysis, positive associations with the disease diffusion were found for seven factors, and the top three drivers are intersection with main roads [adjusted hazard ratio (HR) = 15.45, 95% *CI*: 2.11–113.26, *P* = 0.007], intersection with railways (adjusted HR = 2.33, 95% *CI*:1.37–3.97, *P* = 0.002) and elevation (adjusted HR = 2.40, 95% *CI*: 1.47–3.91, *P* < 0.001). A higher coverage of cropland seemed to have impeded the disease diffusion (adjusted HR = 0.51, 95% *CI*: 0.27–0.95, *P* = 0.034). We overlaid the land coverage and transportation networks with the spatiotemporal distribution of the first reported cases in each space unit (Fig. 2b). In the Arabian Peninsula, intersection with major roads and railroads was clearly associated with earlier invasion.

**Table 3:** Survival analyses on the time to the first reported Middle East respiratory syndrome case in each space unit, September 2012–June 2020

Predictors	Univariable			Multivariable		
	Hazard ratio	95% *CI*	*P-*value	Hazard ratio	95% *CI*	*P-*value
Population density(10/km^2^)	1.00	0.99–1.00	0.44			
Camel density (10/km^2^)	1.74	1.35–2.23	< 0.001			
Elevation (km)	0.99	0.66–1.46	0.94	2.40	1.47–3.91	< 0.001
Meteorological variable						
BIO1	1.58	1.48–1.68	< 0.001	1.38	1.25–1.52	< 0.001
BIO2	1.97	1.74–2.23	< 0.001	1.28	1.12–1.46	< 0.001
BIO3	1.04	1.01–1.07	0.012			
BIO4	1.003	1.002–1.005	< 0.001			
BIO5	1.36	1.30–1.43	< 0.001			
BIO12	0.78	0.74–0.83	< 0.001			
Percentages of land cover (10%)						
Cropland	0.27	0.14–0.49	< 0.001	0.51	0.27–0.95	0.034
Shrubland	0.20	0.07–0.58	0.003			
Bare land	1.49	1.39–1.59	< 0.001	1.13	1.03–1.25	0.014
Intersect with railway	3.76	2.35–6.01	< 0.001	2.33	1.37–3.97	0.002
Intersect with main road	59.53	8.27–428.25	< 0.001	15.45	2.11–113.26	0.007
Number of hospitals (10)	1.28	1.15–1.37	< 0.001	1.19	1.04–1.44	0.010

*BIO1* annual average temperature, *BIO2* average diurnal range of temperature, *BIO3* isothermality, *BIO4* temperature seasonality, *BIO5* max temperature of warmest month, *BIO12* Annual precipitation

Turkey and Iran are excluded in this analysis. Space unit is the third-level administrative area (county) in Saudi Arabia and the second-level administrative area elsewhere

### Phylogeny and phylogeographic analysis of whole-genome sequences

In total, 499 MERS-CoV full-genome sequences were obtained from GenBank, including 251 sequences from human patients, 237 from camel, seven from bat, three from hedgehog, and one from *Lama glama* (llama). These sequences were collected between 2011 and 2019 from 15 countries, and 90.0% of them were from Middle East. IQ-Tree selected the GTR and FreeRate with ten categories as the best substitution model for these sequences.

#### Phylogeny

**Fig. 3:**
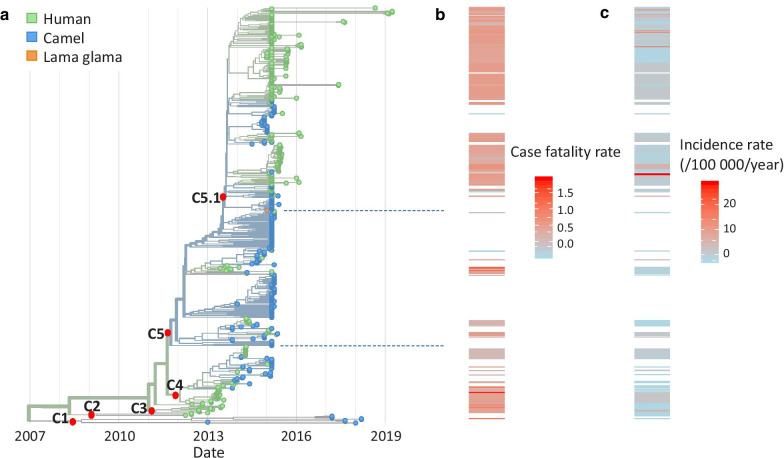
Phylogeny of 489 MERS-CoV whole-genome sequences and potential association of subclades with case fatality. **a** Time-resolved phylogeny. The colors indicate host states of tree tips or inferred host states of the internal nodes. Case fatality rate **b** and incidence rate **c** were associated with the sequences according to their sampling date and location. Movie 1. Spatiotemporal migration dynamics of clade C of MERS-CoV using Nextstrain. https://nextstrain.org/community/wqshi/merse

An initial analysis showed that sequences from bat and hedgehog form a separate clade distant from the main clade of sequences from humans, camels and *Lama glama* (Additional file 1: Figure S5a), confirming that camel is the major zoonotic reservoir of MERS-CoV for spillover to human. We excluded the ten sequences from bat and hedgehog from subsequent analyses. The main clade of human, camel and llama strains, named clade C, contains five subclades numbered C1–C5, with C5 being the largest subclade with 398 sequences (Fig. 3, Additional file 1: Figure S5b). Overall, sequences from human and camel mixed throughout the whole tree, indicating multiple introduction events from camel to human. Nevertheless, the human and camel sequences sampled after 2016 in our database were genetically distant from each other. The root ancestor of clade C, dated back to January 2007 (confidence interval: April 2006–September 2008), was 49.3% likely from camel and 50.7% likely from human. The case mortality rates differed between clades in the phylogenetic tree. C5 was associated with a higher mortality rate than other clades (a difference of 1% in CFR, *P*-value = 2.0 × 10^–4^, Fig. 3). A sub-clade of C5, C5.1, showed an even higher mortality rate than clades C1–C4 (a difference of 4% in CFR, *P*-value < 2.2 × 10^–16^). Compared to subclades C1–C4, we found non-synonymous mutations in regions encoding the ORF3 protein (P86L) and the NS4B protein (M6T) among the C5.1 sequences as well as in regions encoding the 1AB protein (S6737N), the NS4A protein (P106S), and the Membrane protein (V69I) among other C5 sequences.

#### Phylogeographic dynamics

The spatiotemporal transmission pattern of clade C was characterized by intense local migration within the Middle East and occasional long distance exportation (Movie 1; Additional file 1: Figure S6). The top three most likely locations of the inferred root ancestor were Riyadh of Saudi Arabia, the Nile Delta region and Jordan with posterior probabilities of 31%, 17% and 12%, respectively. Riyadh appeared to be the major source exporting infections both locally and internationally. It was estimated with 99% posterior probability as the location of common ancestral node for subclades C3, C4, and C5 which cover 97.5% of the collected sequences. Early exportation of the virus to Egypt and Jordan likely occurred before 2010. The circulation of MERS-CoV among dromedary camels in East Africa possibly started before 2010. The model inferred migration of the virus from Egypt to Ethiopia during 2011–2013 and subsequently to Kenya during 2014–2017, partly supported by a serological study conducted in Egypt in 2013 that found both domestic dromedary camels and those imported from Ethiopia were seropositive [29]. Intense migration of the virus from Riyadh towards local cities in Saudi Arabia, Abu Dhabi in UAE and Europe started during 2011–2012. Abu Dhabi soon joined Riyadh as the second hub exporting the virus to other Middle East cities as well as to Europe. The opportunistic exportation events from the Middle East to the United States in 2014 and to East Asia in 2015 were correctly captured by the model.

#### Positive selection

To identify key genetic sites affecting the transmissibility of MERS-CoV from animals to human, we performed positive selection analysis on the collected sequences. Based on the phylogenetic tree, we focused on two branches with potentially high selection pressure (Additional file 1: Figure S5a), the branch separating the hedgehog-related clade from the clades among bat, camel and human (branch A), and the one separating the bat-related sequences from clade C among camel and human (branch B). We tested whether specific sites underwent positive selection along branches A or B using the branch-site test model implemented in the codeML program of the PAML package. For branch A, we identified three proteins (NS3, 1AB polyprotein and nucleoprotein) and 59 sites in the 1AB polyprotein under positive selection (Additional file 1: Table S8). For branch B, we identified two different proteins (ORF8b and spike glycoprotein) and eight sites in the spike glycoprotein under positive selection. Three of the eight spike glycoprotein sites, 77:Y, 486:H and 636:Q have not been previously reported. No positive selection was detected in the spike glycoprotein for branch A.

## Discussion

Combining updated case data, genetic data and relevant socioenvironmental data, we provided an in-depth analysis of the epidemiology of MERS and genetic evolution of MERS-CoV in the most affected regions. Countries in the Middle East, particularly the Saudi Arabia, continue to be the epicenter of MERS, with frequent animal-to-human spillovers in the region and sporadic exportation of human cases to other continents. Patients ≥ 65 years old or with underlying conditions had a significantly higher risk of death. The effect of animal contact on the risk of death depends on both age and sex. Transportation network was the leading driver for the spatial diffusion of the disease.

We identified marked differences in demographic and clinical features of cases between the two transmission modes, human-to-human vs animal-to-human, e.g., cases with animal contact tended to be older, more likely to be male and symptomatic, and more likely to have underlying conditions and longer delay of diagnosis. The two modes also differ in seasonality. Animal-to-human transmission events occurred mainly between January and March, and human-to-human transmission peaks subsequently from April to June [24]. This temporal order reflects the importance of blocking animal-to-human spillover in early spring. Preventive measures such as educational campaigns and advocating personal protection equipment among workers of camel farms and trading posts can be used to reduce zoonotic infection in the high-risk season.

Risk factors for case fatality in our analyses includes the elderly (≥ 65 years old), male sex, Middle Eastern residents, underlying diseases, and animal exposure, which are in line with previous findings [31]. Our analyses further revealed that the dependence of the risk of fatality on animal contact history varied by age group and sex. animal contact history was a risk factor for death in female patients < 65 years old but was protective in male patients ≥ 65 years old. This observation could be explained by the possibility that, in the Middle East, long-term exposure to dromedary camels has built immunity in older males, and opportunistic exposure of fully susceptible females, especially those younger than 65 years, could be more lethal. This possibility is partly supported by the higher seroprevalence in older males than other age and sex groups [32]. Special attention should be paid and timely treatment should be provided to young female and old male patients upon admission or diagnosis to prevent severe adverse outcomes.

We found the road and railway traffic network played an important role in the rapid regional dispersion of MERS. While the virus has not gained efficient human-to-human transmissibility, the frequent domestic and international migrations of infected humans and animals within the Middle East and between the Middle East and the rest of the world are imposing a sustained risk of viral adaption to human immune system, similar to SARS-CoV-2. The outbreak involving 186 cases and 36 deaths in the Republic of Korea highlighted this threat [13]. Surveillance and screening of infected travelers at transportation hubs such as international airports, are needed, especially in areas with frequent travelers from and to the epidemic areas. Avoiding congestion of dromedary herds at watering sites and during their transportation is likely an effective control measure to reduce the circulation of the virus in its natural animal reservoir and thereby to reduce the chance of spillover to humans. Vaccination of dromedary camels could be a better option depending on the development progress and availability of effective vaccines [33].

Our phylogenetic analysis estimated that the inferred root ancestor was 49.3% likely from camel and 50.7% likely from human, different from another study that reported camel to be the sole possible host of ancestral root [20]. This gap might result from the differences in model structures and assumptions as well as in sequence samples. In a sensitivity analysis, we down-sampled sequences after 2014 and estimated camel to be the host of root ancestor with a posterior probability of 99.4%, suggesting existing sequences may not represent the true spatiotemporal distribution of the underlying viral population and hence may lead to biased and unstable estimation. Likewise, we also caution the interpretation of the potential association of clade C5 with a higher mortality rate, which needs to be verified in the future.

Our phylogeographic analysis revealed the dissemination history of MERS-CoV both within the Middle East and from the peninsula to other continents. The spatiotemporal transmission pattern of clade C was basically consistent with the spatiotemporal distribution of cases at the individual level and early diffusion at the population level. The fact that Abu Dhabi of UAE became the second hub for international exportation of the virus is highly alarming, indicating the necessity of screening MERS-CoV-infected travelers at international airports. However, there still exists inconsistency between some fine details of model-estimated migration trajectories and epidemiological data. For example, the patient who traveled from Saudi Arabia to Chicago was shown by the model as coming from UAE, and the first exportation to East Asia was misplaced at Southern China instead of Republic of Korea [34, 35]. However, such inconsistency in fine details does not alter the big picture of intense local migration in the Middle East and early circulation in the northern and eastern Africa. Future phylogeographic analysis should incorporate prior epidemiological information if such methodology becomes available.

We identified eight amino acid positions in the spike glycoprotein potentially associated with positive selection, three of which are novel sites. The spike glycoprotein is known to mediate viral entry and affect the host range of MERS-CoV and had been previously reported to be under positive selection [36]. The novel sites found in this study may provide research directions for potential targets for development of antivirals and vaccines against MERS-CoV [37].

A few limitations of the study are worth mentioning. First, the passive surveillance systems of MERS in most countries only capture patients who sought medical care, while patients with subclinical or asymptomatic infections are usually missed. This surveillance bias may affect our assessment of the relationship between animal contact history and asymptomatic infection. Second, not all relevant risk drivers or confounders were considered in our diffusion analysis and the regression model for case fatality due to lack of data, e.g., the density of dromedary camels and the number of healthcare facilities. Finally, the potential sampling bias in the whole-genome sequences could have affected the phylogenetic, phylogeographic and positive selection analyses, which may not be fully addressable by down-sampling.

## Conclusions

Despite its current incompetence for human-to-human transmission, MERS-CoV has successfully expanded its enzootic range throughout the Middle East, North and East Africa, and West and Southwest Asia, and imposing imminent pandemic threat through genetic mutation or recombination with other human coronaviruses, especially given the widespread of SARS-CoV-2. Active surveillance of adapting mutants among human patients and animal reservoir, as well as screening of infected travelers at transportation hubs such as international airports, are urgently needed. While the rapid development of SARS-CoV-2 vaccines shed lights on how to advance MERS-CoV vaccine candidates, nonpharmaceutical interventions and animal vaccines should be planned ahead to delay or block the adaption of MERS-CoV at the source.

## Supplementary Information

**Additional file 1: **Additional tables and figures.

## Data Availability

Please contact author for data requests.

## Abbreviations

CDS: Coding sequences
CFR: Case fatality rate
*CI*: Confidence Interval
FAO: Food and Agriculture Organizationon
HR: Hazard ratio
IQR: Interquartile range
MERS: Middle East respiratory syndrome
MERS-CoV: Middle East respiratory syndrome coronavirus
*OR*: Odds ratio
ORF: Open reading frame
OTC: Time from disease onset to confirmation
WHO: World Health Organization
