# Supplementary material for: Epidemiology and evolution of Middle East respiratory syndrome coronavirus, 2012–2020
Source: Infect Dis Poverty. 2021 May 8;10:66. doi: 10.1186/s40249-021-00853-0 (PMC8105704; doi:10.1186/s40249-021-00853-0)
Supplement: Supplementary file 1 — Additional file 1: Additional tables and figures. [file 40249_2021_853_MOESM1_ESM.doc]

**Supplementary Appendix:**

A.R. Zhang, et al. Epidemiology and Evolution of Middle East Respiratory Syndrome Coronavirus, 2012‒2020

**Data collection and management**

We assembled a comprehensive database of all confirmed MERS cases up to 1 June, 2020, mainly extracted from the official reports of the World Health Organization (WHO, https://www.who.int/emergencies/mers-cov/en/). WHO requests Member States to report all cases infected with MERS-CoV together with information about their exposure, testing results, and clinical outcomes. According to the WHO technical guidance, a confirmed case is a person with laboratory confirmation of MERS-CoV infection, irrespective of clinical symptoms. To be admissible to our analysis, a MERS case record must include the basic demographic information (gender, age, reporting country, city of residence, being a health care worker or not, baseline chronic conditions), dates of critical events (such as symptoms onset, first hospitalization and laboratory confirmation), and exposure information (whether exposed to animal or raw animal production, or exposed to confirmed MERS cases). The WHO dataset contains 1981 confirmed cases with complete information. For those cases with missing key information, we sought information from other public sources, including 457 case reported by the health departments of major affected countries (mainly the Ministry of Health of Saudi Arabia), and 12 cases from relevant news reports and articles. We matched and cross-validated cases between sources according to age, sex and city of cases as well as other available information. Cases with most key information missing even after cross-validation were excluded. In total, 2450 lab-confirmed cases were admissible to our study.

Data on socioenvironmental variables possibly contributing to the diffusion and persistence of MERS were collected. Sources of different kinds of data were shown in Table S1. Population density was derived from Socioeconomic Data and Applications Centre (SEDAC) of the International Geographic Science Information (http://sedac.ciesin.columbia.edu/gpw/credits.jsp). Camel density during 2012 to 2020 were obtained from Office International Des Epizooties (OIE, http://www.oie.int/wahis_2/public/wahid.php/Countryinformation/Animalpopulation). A recent data indicated that primary MERS cases were more likely to occur on cold and dry days in Saudi Arabia.[1] So monthly meteorological data during the study period, including average temperature and relative humidity, were obtained from National Oceanic and Atmospheric Administration (NOCC, http://www.ncdc.noaa.gov/). Based on them, 19 ecoclimatic variables (BIO01‒19) were created and applied in the machine learning models to avoid causing effect bias due to the heterogeneity of months in different seasons among a wide latitude range in the world.[2] Elevation value was derived from the shuttle radar topography mission (SRTM) 90m DEM Digital Elevation Database (http://srtm.csi.cgiar.org). Land cover data were derived from the European Aviation Administration (http://ionial.esrin.esa.int). Percentage coverage of cropland, forest, grassland, wetland, waterbody, artificial surfaces, bareland, and ice areas were extracted and summarized at the study-needed spatial resolution. To explore the relationship between the spatial spread of MERS and socio-environmental factors, the following data were also collected at the county level from the OpenStreetMap project (http://download.geofabrik.de/): transportation (major roads and railways) and locations of hospitals. For population density, elevation and land cover, those collected as raster digital maps were converted to the study’s geographic projection first, and were then extracted and summarized into a format at the study-needed resolution based on the boundary data in ArcGIS 10.5 (Esri Inc, Redlands, CA, USA). The boundary data were obtained from the GADM database of Global Administrative Areas version 2.0 (http://www.gadm.org). For the phylodynamic and phylogeographic analyses, full-genome sequences (>30,000bp) of MERS-CoV with background information including isolation year, host, and location were retrieved from GenBank (https://www.ncbi.nlm.nih.gov/genbank/, as date of 1 June 2020). Sequences without collection date, country or host labels were filtered out.

All data were cleaned and processed in R 3.6.2 with appropriate data quality control. Finally, a total of 34 socioenvironmental variables that may affect spatial diffusion of the disease were extracted for each second-level administrative area (third-level administrative area for Saudi Arabia) from the database (Table S3).

**Smoothed case data associated with the phylogenetic tree**

In the phylogenetic tree, both mortality rate and incidence rate were associated with tree tips (MERS-CoV sequences) based on collection year and location. For location, we used the rates of secondary administrative unit where the collection site was located, and rates of country if no specific address information. In order to reduce the bias caused by the uneven distribution of data, we smoothed the data for Saudi Arabia, the country with the highest MERS case number, using Kriging interpolation method.

**Reference**

1 Gardner EG, Kelton D, Poljak Z, Van Kerkhove M, von Dobschuetz S, Greer AL. A case-crossover analysis of the impact of weather on primary cases of Middle East respiratory syndrome. *BMC Infect Dis* 2019; **19:** 113.

2 Hijmans RJ, Cameron SE, Parra JL, Jones PG, Jarvis A. Very high resolution interpolated climate surfaces for global land areas. *Int J Climatol* 2005; **25:** 1965–78.

**Table S1: Description of data sources in this study.**

| Variable | Source | Note | Website |
| --- | --- | --- | --- |
| Human MERS cases | World Health Organization, WHO | 1,981 cases with individual information | https://www.who.int/emergencies/mers-cov/en/ |
|  | Ministry of Health, Kingdom of Saudi Arabia | 457 cases added | https://www.moh.gov.sa/en/CoronaNew/PressReleases/Pages/default.aspx |
|  | Other news reports or articles | 12 cases added |  |
| Population density | Socioeconomic Data and Applications Centre (SEDAC) of the International Geographic Science Information | A raster digital map with a resolution of 30 arc seconds | http://sedac.ciesin.columbia.edu/gpw/credits.jsp |
| Camel density | Office International Des Epizooties, OIE |  | <http://www.oie.int/wahis_2/public/wahid.php/Countryinformation/Animalpopulation> |
|  | Food and Agriculture Organization of the United Nations, FAO | FAOSTAT country-level camel population data | http://www.fao.org/faostat/en |
| Meteorological data | National Oceanic and Atmospheric Administration, NOCC | Including average temperature and relative humidity, which 19 ecoclimatic variables (BIO01‒19) were created based on. | http://www.ncdc.noaa.gov/ |
| Elevation | The shuttle radar topography mission (SRTM) 90m DEM Digital Elevation Database |  | http://srtm.csi.cgiar.org |
| Land cover | European Aviation Administration | A raster digital map with a resolution of 300m | http://ionial.esrin.esa.int |
| Transportation | The OpenStreetMap project | Including railways and main roads | http://download.geofabrik.de/ |
| Locations of hospitals | The OpenStreetMap project |  | http://download.geofabrik.de/ |
| Map data | GADM database of Global Administrative Areas version 2.0 | Boundary data | http://www.gadm.org |
| Sequences of MERS-CoV | Genbank | 499 full-genome sequences (>30,000bp) | https://www.ncbi.nlm.nih.gov/genbank/ |

**Table S2: Country-specific reported numbers of confirmed MERS cases and case clusters from 2012 to 2020. The major transmission type is determined for each country by exposure history of cases and literature.**

| Reporting country | Number of  reported cases | Number of  reported clusters | Transmission Types |
| --- | --- | --- | --- |
| Jordan | 28 | 4 | I |
| Oman | 24 | 2 | I |
| Qatar | 27 | 3 | I |
| Saudi Arabia | 2,048 | 123 | I |
| United Arab Emirates | 93 | 11 | I |
| Kuwait | 4 | 0 | II |
| France | 2 | 1 | III |
| Iran | 6 | 1 | III |
| Netherlands | 2 | 1 | III |
| Republic of Korea | 186 | 1 | III |
| Tunisia | 2 | 1 | III |
| United Kingdom | 4 | 1 | III |
| United States of America | 3 | 1 | III |
| Algeria | 2 | 0 | IV |
| Austria | 2 | 0 | IV |
| Bahrain | 1 | 0 | IV |
| China | 1 | 0 | IV |
| Egypt | 1 | 0 | IV |
| Germany | 1 | 0 | IV |
| Greece | 1 | 0 | IV |
| Italy | 1 | 0 | IV |
| Lebanon | 2 | 0 | IV |
| Malaysia | 2 | 0 | IV |
| Philippines | 2 | 0 | IV |
| Thailand | 3 | 0 | IV |
| Turkey | 1 | 0 | IV |
| Yemen | 1 | 0 | IV |
| Total | 2,450 | 150 | .. |

a. (I) Zoonotic transmission plus human-to-human transmission, (II) Zoonotic transmission without human-to-human transmission, (III) Case importation plus human-to-human transmission, and (IV) Case importation without human-to-human transmission

**Table S3: Description of 34 socioenvironmental variables potentially influencing spatial diffusion of MERS.**

| Variable | Description | Type |
| --- | --- | --- |
| BIO01 | Annual mean temperature (℃) | Continuous |
| BIO02 | Mean diurnal range (Mean of monthly (max temp-min temp)) (℃) | Continuous |
| BIO03 | Isothermality (BIO02/BIO07)(*100) | Continuous |
| BIO04 | Temperature seasonality (standard deviation*100) | Continuous |
| BIO05 | Max temperature of warmest month (℃) | Continuous |
| BIO06 | Min temperature of coldest month (℃) | Continuous |
| BIO07 | Annual range of temperature (BIO05-BIO06) (℃) | Continuous |
| BIO08 | Mean temperature of wettest quarter (℃) | Continuous |
| BIO09 | Mean temperature of driest quarter (℃) | Continuous |
| BIO10 | Mean temperature of warmest quarter (℃) | Continuous |
| BIO11 | Mean temperature of coldest quarter (℃) | Continuous |
| BIO12 | Annual precipitation (mm) | Continuous |
| BIO13 | Precipitation of wettest month (mm) | Continuous |
| BIO14 | Precipitation of driest month (mm) | Continuous |
| BIO15 | Precipitation seasonality(Coefficient of variation) | Continuous |
| BIO16 | Precipitation of wettest quarter (mm) | Continuous |
| BIO17 | Precipitation of driest quarter (mm) | Continuous |
| BIO18 | Precipitation of warmest quarter (mm) | Continuous |
| BIO19 | Precipitation of coldest quarter (mm) | Continuous |
| Cropland | Percentage coverage of cropland (%) | Continuous |
| Forest | Percentage coverage of forest land (%) | Continuous |
| Grassland | Percentage coverage of grassland (%) | Continuous |
| Shrubland | Percentage coverage of shrubland (%) | Continuous |
| Wetland | Percentage coverage of wetland (%) | Continuous |
| Built-up land | Percentage coverage of built-up land (%) | Continuous |
| Bare land | Percentage coverage of bare land (%) | Continuous |
| Water body | Percentage coverage of water body (%) | Continuous |
| Ice land | Percentage coverage of ice land (%) | Continuous |
| Popular density | Density of human population (100 person per km^2^) | Continuous |
| Camel density | Density of camel (1 heads per km^2^) | Continuous |
| Elevation | Average altitude (m) | Continuous |
| Railway | Having railways or not | Binary |
| Main road | Having main roads or not | Binary |
| Hospital | Number of hospitals | Continuous |

**Table S4: Comparison of characteristics between cases with animal contact and those without.**

|  | No. of cases (%) | | p-value^a^ |
| --- | --- | --- | --- |
|  | With Animal Contact | Without Animal Contact |  |
| No. of confirmed cases | 356 | 1097 |  |
| Female | 23 (6.46) | 461 (42.02) | <0.001^*^ |
| Age, years (median, IQR) | 59 (47‒70) | 45 (32‒61) | <0.001^**^ |
| Deaths (CFR, %) | 125 (35.11) | 267 (24.34) | <0.001^*^ |
| Healthcare worker | 1 (0.28) | 284 (25.89) | <0.001^*^ |
| Asymptomatic | 5 (1.40) | 177 (16.13) | <0.001^*^ |
| Underlying condition | 274 (76.97) | 363 (33.09) | <0.001^*^ |
| Time from disease onset to diagnosis, days (median, IQR) | 6.5 (4‒9) | 3 (2‒6) | <0.001^**^ |
| Time from disease onset to death, days (median, IQR) | 11.5 (8-18) | 9 (5‒15) | <0.001^**^ |
| Year^b^ |  |  | 0.001^***^ |
| 2012-2013 | 9 (9.1) | 90 (90.9) |  |
| 2014 | 33 (11.0) | 267 (89.0) |  |
| 2015 | 55 (11.2) | 437 (88.8) |  |
| 2016 | 73 (47.4) | 81 (52.6) |  |
| 2017 | 74 (44.8) | 91 (55.2) |  |
| 2018 | 47 (58.0) | 34 (42.0) |  |
| 2019 | 53 (38.3) | 87 (61.7) |  |
| 2020 | 11 (52.4) | 10 (47.6) |  |

a. * Variables using χ² test, ** Variables using Kruskal-Wallis test, *** Cochran-Armitage trend test.

b. Row percentages are shown

**Table S5: Effects of age group on the risk of death conditional on sex and animal contact history in a multivariable logistic regression.**

| Sex | Animal contact | Age group | Death/Total | CFR(95% CI) | Adjusted OR  (95% CI) | p-value |
| --- | --- | --- | --- | --- | --- | --- |
| Male | With | ≥65 | 56/123 | 45.53 (36.73‒54.33) | 1.64 (1.03‒2.62) | 0.038 |
|  |  | <65 | 59/210 | 28.1 (22.02‒34.17) | 1.00 | - |
|  | Without | ≥65 | 92/136 | 67.65 (59.78‒75.51) | 5.65 (3.75‒8.50) | <0.001 |
|  |  | <65 | 95/500 | 19 (15.56‒22.44) | 1.00 | - |
|  | Unknown | ≥65 | 135/224 | 60.27 (53.86‒66.68) | 2.54 (1.84‒3.50) | <0.001 |
|  |  | <65 | 167/506 | 33 (28.91‒37.10) | 1.00 | - |
| Female | With | ≥65 | 4/8 | 50 (15.35‒84.65) | 3.06 (1.59‒5.86) | 0.001 |
|  |  | <65 | 6/15 | 40 (15.21‒64.79) | 1.00 | - |
|  | Without | ≥65 | 48/83 | 57.83 (47.21‒68.46) | 10.52 (6.46‒17.11) | <0.001 |
|  |  | <65 | 32/378 | 8.47 (5.66‒11.27) | 1.00 | - |
|  | Unknown | ≥65 | 57/80 | 71.25 (61.33‒81.17) | 4.73 (2.96‒7.56) | <0.001 |
|  |  | <65 | 51/187 | 27.27 (20.89‒33.66) | 1.00 | - |

**Table S6: Effects of sex on the risk of death conditional on age group and animal contact history in a multivariable logistic regression.**

| Age group | Animal contact | Sex | Death/Total | CFR (95% CI) | Adjusted OR  (95% CI) | p-value |
| --- | --- | --- | --- | --- | --- | --- |
|  |  |  |  |  |  |  |
| ≥65 | With | Male | 56/123 | 45.53 (36.73‒54.33) | 0.45 (0.17‒1.19) | 0.11 |
|  |  | Female | 4/8 | 50 (15.35‒84.65) | 1.00 | - |
|  | Without | Male | 92/136 | 67.65 (59.78‒75.51) | 1.26 (0.78‒2.02) | 0.34 |
|  |  | Female | 48/83 | 57.83 (47.21‒68.46) | 1.00 | - |
|  | Unknown | Male | 135/224 | 60.27 (53.86‒66.68) | 0.73 (0.47‒1.16) | 0.19 |
|  |  | Female | 57/80 | 71.25 (61.33‒81.17) | 1.00 | - |
| <65 | With | Male | 59/210 | 28.1 (22.02‒34.17) | 0.83 (0.32‒2.16) | 0.71 |
|  |  | Female | 6/15 | 40 (15.21‒64.79) | 1.00 | - |
|  | Without | Male | 95/500 | 19 (15.56‒22.44) | 2.34 (1.57‒3.49) | <0.001 |
|  |  | Female | 32/378 | 8.47 (5.66‒11.27) | 1.00 | - |
|  | Unknown | Male | 167/506 | 33 (28.91‒37.10) | 1.37 (0.96‒1.95) | 0.082 |
|  |  | Female | 51/187 | 27.27 (20.89‒33.66) | 1.00 | - |

**Table S7: Effects of animal contact history on the risk of death conditional on sex and age group in a multivariable logistic regression.**

| Sex | Age group | Animal contact | Death/Total | CFR (95% CI) | Adjusted OR  (95% CI) | p-value |
| --- | --- | --- | --- | --- | --- | --- |
| Male | ≥65 | With | 56/123 | 45.53 (36.73‒54.33) | 0.31 (0.18‒0.51) | <0.001 |
|  |  | Without | 92/136 | 67.65 (59.78‒75.51) | 1.00 | - |
|  |  | Unknown | 135/224 | 60.27 (53.86‒66.68) | 0.63 (0.41‒0.98) | 0.039 |
|  | <65 | With | 59/210 | 28.1 (22.02‒34.17) | 1.05 (0.71‒1.57) | 0.79 |
|  |  | Without | 95/500 | 19 (15.56‒22.44) | 1.00 | - |
|  |  | Unknown | 167/506 | 33 (28.91‒37.10) | 1.41 (1.04‒1.90) | 0.025 |
| Female | ≥65 | With | 4/8 | 50 (15.35‒84.65) | 0.86 (0.31‒2.43) | 0.78 |
|  |  | Without | 48/83 | 57.83 (47.21‒68.46) | 1.00 | - |
|  |  | Unknown | 57/80 | 71.25 (61.33‒81.17) | 1.08 (0.65‒1.82) | 0.76 |
|  | <65 | With | 6/15 | 40 (15.21‒64.79) | 2.97 (1.10‒7.98) | 0.031 |
|  |  | Without | 32/378 | 8.47 (5.66‒11.27) | 1.00 | - |
|  |  | Unknown | 51/187 | 27.27 (20.89‒33.66) | 2.41 (1.54‒3.77) | <0.001 |

**Table S8: Identified proteins and amino acid sites under positive selection.**

| Protein | Foreground | lnL MA1 | lnL MA | *p*-value | Adjust *p*-value | Identified sites^§^ |
| --- | --- | --- | --- | --- | --- | --- |
| NS3 | Bat, human, camel | -1883.9 | -1887.4 | 0.008 | 0.03 | Not identified |
| Nucleoprotein | Bat, human, camel | -5762.3 | -5767.4 | 0.001 | 0.01 | Not identified |
| 1AB | Bat, human, camel | -96465.2 | -96484.3 | <0.001 | <0.001 | 109:S*,111:S*,112:A*,214:A*,248:L*,261:P*,301:K*,302:W*,353:Q*,369:E*,418:D*,421:S*,483:S*,664:A*,673:A*,756:Q*,773:G*,941:P*,942:C*,1104:K*,1249:Y*,1308:S*,1309:A*,1317:K*,1352:S*,1420:N*,1448:C*,1513:C*,1543:A*,1568:K*,1800:S*,1817:V*,2079:A*,2241:S*,2252:S*,2322:S*,2358:C*,2643:S*,2833:I*,2989:A*,3040:F*,3117:G*,3293:A*,3317:K*,3474:R*,3527:Q*,3699:C*,3721:V*,3778:C**,3798:D*,4077:N*,4300:A*,4659:Q*,5151:K*,5390:S*,5924:S*,5984:R*,6280:R*,6826:C* |
| ORF8b | Human, camel | -1354.7 | -1359.2 | 0.003 | 0.02 | Not identified |
| Spike | Human, camel | -22959.2 | -22963.4 | 0.004 | 0.02 | 77:Y*,270:Y*,436:N**,479:L*,486:H*,521:N*,636:Q*,775:S** |

§ Sites with post posterior probability (PP) > 0.95 are shown (* indicates PP > 0.95, and ** indicates PP > 0.99). Likelihood ratio test was used to obtain the *P*-value of alternative MA1 model.

**Figure S1: Distribution of MERS cases clusters in the world from September 2012 to June 2020.** The size of the circle represents the number of clusters that occurred at the location.

**
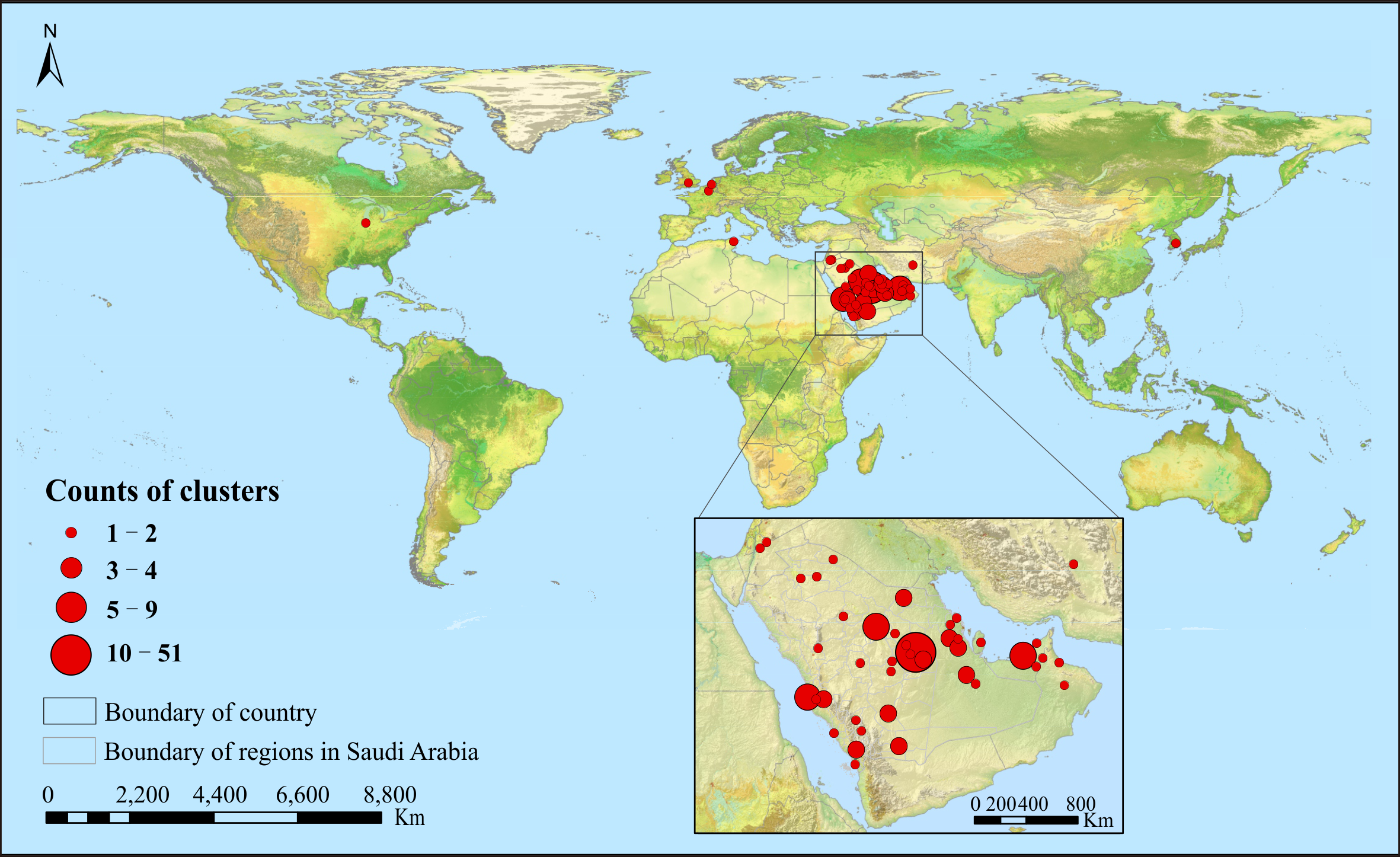
**

**Figure S2: Spatial-temporal distribution of clusters of MERS cases in the world from September 2012 to June 2020.** (A) Distribution of clusters of MERS cases in the world. The size of each circle represents the cumulative number of cases in all clusters at the location. (B) Distribution of clusters in the Middle East by year. Each cluster is marked with a circle, size and color representing the number of cases and case type (primary vs. non-primary), respectively.


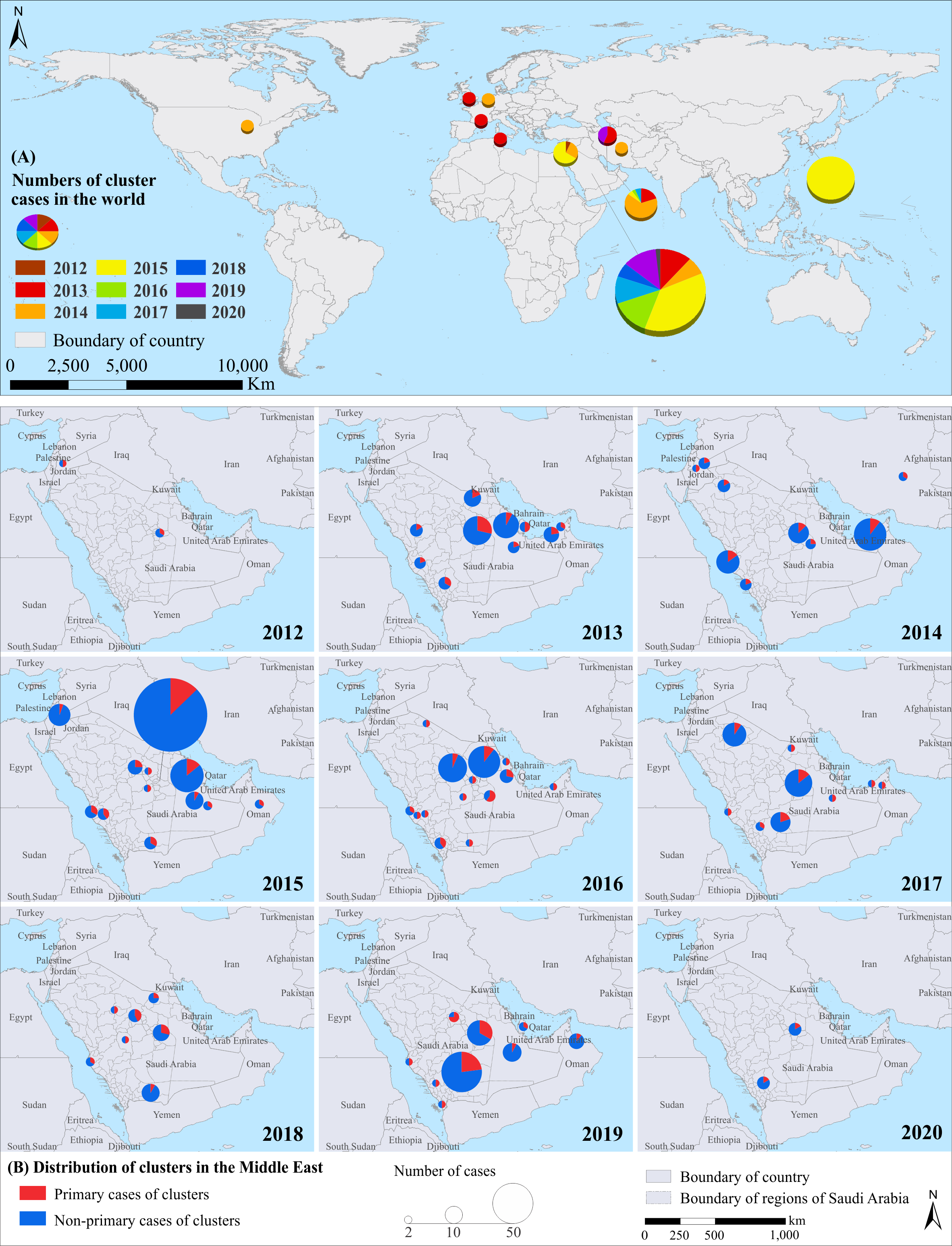


**Figure S3: Yearly and seasonal trends of MERS epidemic in the world and in selected countries from September 2012 to June 2020.** The seasonality was represented by the radar diagram. The 12 months follow the clockwise direction, and the radius represents time (left). Weekly numbers of symptom onsets of confirmed MERS cases are plotted for the whole world and for the two countries most affected by MERS-CoV, Saudi Arabia and Unite Arab Emirates (right).

**
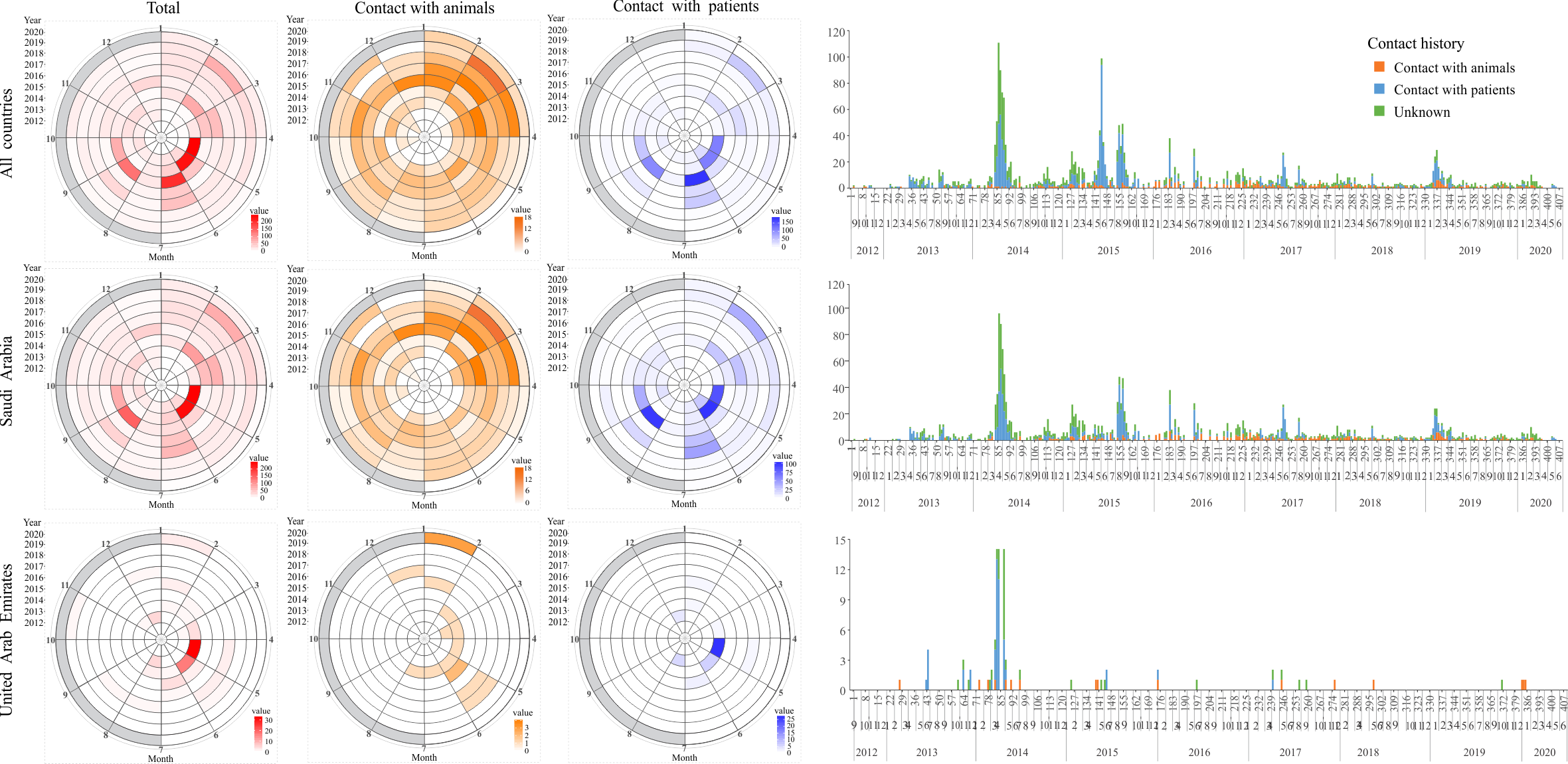
**

**Figure S4: Annual case numbers (bars) and case fatality rates (dotted lines) stratified by patients’ sex (A), age (B), contact history (C) and preexisting comorbidities (D).** Sparse data in 2012 or 2013, as well as data in 2019 or 2020, were merged in all panels to avoid undue impact on the overall trend in mortality.

**
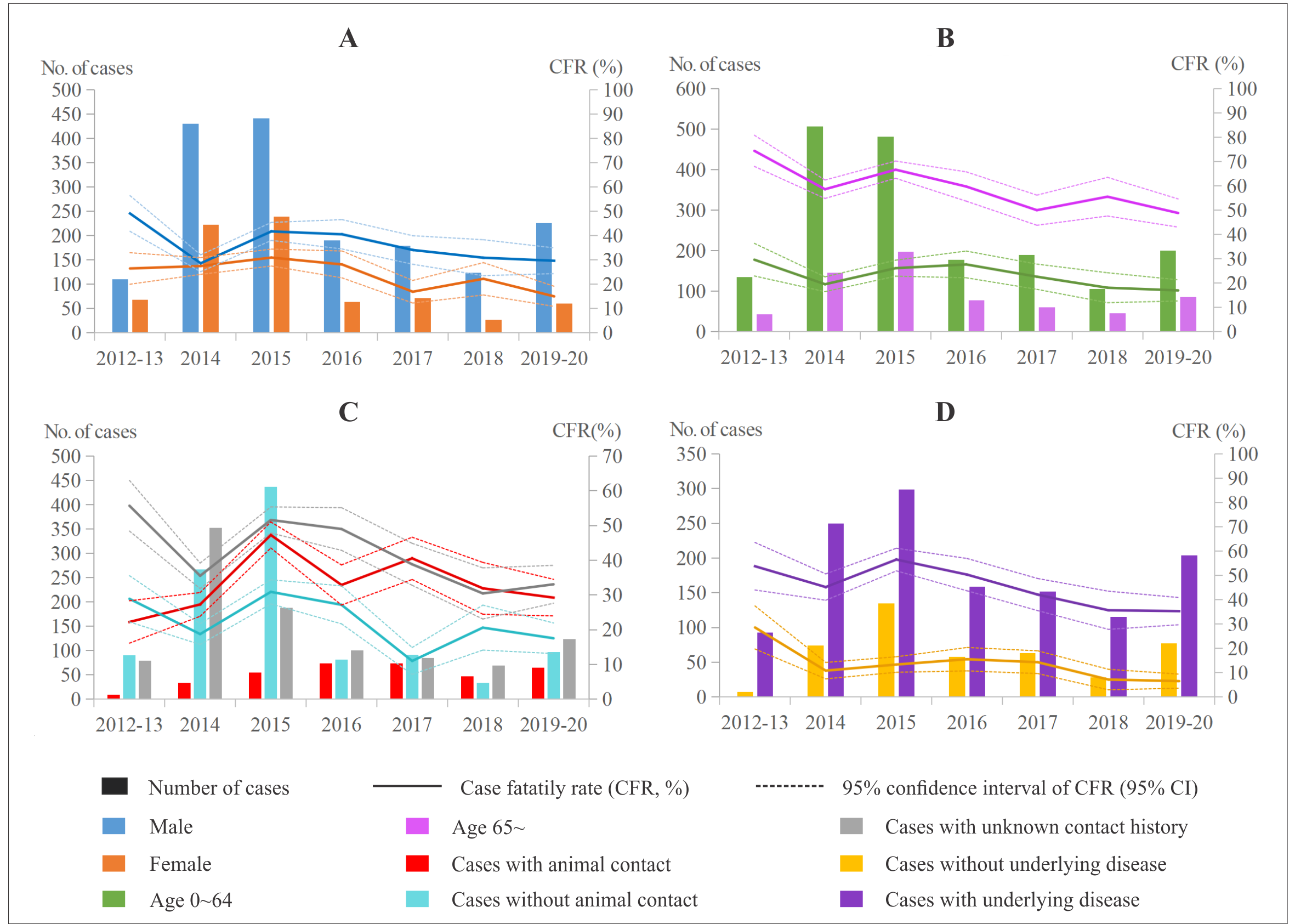
**

**Figure S5: Phylogeny of MERS-CoV sequences.** The left tree (A) was based on sequences collected from camel, hedgehog, human, lama glama and bat. Sequences from camel, human and lama glama clustered into the largest clade. The phylogenetic tree is represented (B) to show the internal structure of the major clade.


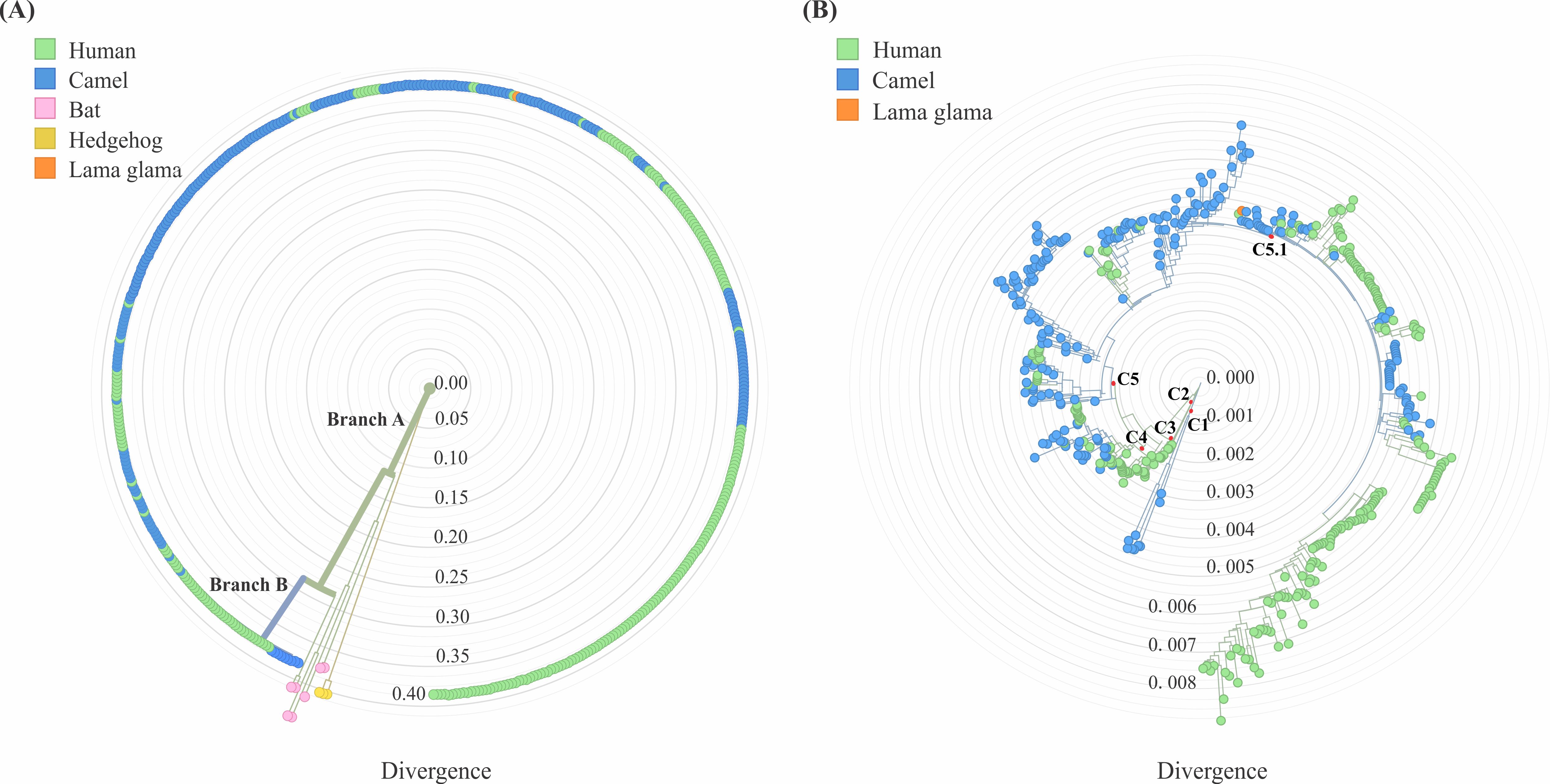


**Figure S6: Migration patterns inferred from MERS-CoV whole-genome sequences using Nextstrain.**

(A) Worldwide migration pattern; (B) Migration pattern in the Middle East where most sequences were sampled. The size of each disc indicates the accumulated number of sequences. Each arc connects a pair of source and destination. Each arc was colored the same as the source location. The dotted lines indicate migration events contradicting epidemiological observation.
